# Editorial: Therapeutic Drug Monitoring in Solid Organ Transplantation

**DOI:** 10.3389/fphar.2021.815117

**Published:** 2021-12-10

**Authors:** Christine E. Staatz, Nicole M. Isbel, Troels K. Bergmann, Bente Jespersen, Niels Henrik Buus

**Affiliations:** ^1^ School of Pharmacy, University of Queensland, Brisbane, QLD, Australia; ^2^ Department of Nephrology, University of Queensland at the Princess Alexandra Hospital, Brisbane, QLD, Australia; ^3^ Department of Clinical Biochemistry and Pharmacology, Odense University Hospital, Odense, Denmark; ^4^ Department of Regional Health Research, University of Southern Denmark, Esbjerg, Denmark; ^5^ Department of Renal Medicine, Aarhus University Hospital, Denmark

**Keywords:** therapeutic drug monitoring, immunosuppressants, solid organ transplantation, personalizing therapy, tacrolimus, mycophenolic acid

Solid organ transplant recipients are reliant upon a combination of lifelong immunosuppressant medicines to maintain functioning of their grafts. Therapeutic drug monitoring (TDM) is a well-established approach for guiding therapy, which involves individualization of drug dosage by maintaining drug concentrations within a pre-defined target range. TDM is routinely performed during treatment with calcineurin- and mTOR inhibitors and is sometimes undertaken during mycophenolate usage. Consensus reports have established optimal TDM practices for these agents (Shipkova et al., 2016; Brunet et al., 2019; Bergan et al., 2021). While few studies have assessed the clinical benefits of TDM in a prospective, randomised manner, several studies have demonstrated a correlation between low immunosuppressant exposure and risk of acute rejection and high immunosuppressant exposure and drug toxicity.

For practical and economic reasons, TDM is frequently based on a single time-point measure, often the morning trough concentration. The time-of-day monitoring is performed may have implications for immunosuppressant medicines dosed more than once-a-day. Fontova et al. reported lower whole blood and intracellular tacrolimus concentrations after the night dose compared to the same morning dose. These results suggest 24-h variations in both the extent and rate of absorption of tacrolimus due to circadian fluctuations in drug metabolising enzymes and efflux transporter proteins. Some transplant centres may estimate the area-under-the-concentration-time curve (AUC) of some immunosuppressant agents, which is likely a more robust overall measure of drug exposure. AUC can be determined through inclusion of a limited number of concentration-time points in a multiple linear regression equation or use of a population pharmacokinetic model applied in a *Maximum a posteriori* Bayesian estimator (Staatz and Tett, 2011; Brooks et al., 2021). While in its infancy machine learning techniques are also starting to be applied to predict AUC (Wollard et al., 2021a; Wollard et al., 2021b).

Most current immunosuppressant dosing and monitoring protocols do not provide individualised recommendations for different patient populations such as the elderly, the obese or pregnant patients. This is highlighted in Cossart et al.’s., review which suggests that elderly kidney transplant recipients may have higher dose-adjusted exposure and/or lower clearance of the calcineurin inhibitors. Elderly recipients have increased risk of both morbidity and mortality due to increased susceptibility to immunosuppressant side effects, particularly cardiovascular disease, infection, and malignancy. More research is required into the optimal exposure target to aim for in different patient cohorts, including those on varying immunosuppressant combinations, with different rejection and side-effect risk profiles and at different time-points after transplantation.

Currently assessment of immunosuppressant exposure is generally based on the total drug concentration measured in whole blood or plasma samples. High-performance liquid chromatography with tandem mass-spectrometry is the preferred assay technique as it provides high sensitivity and specificity (Zhang and Zhang, 2018). Simultaneous measurement of several immunosuppressant agents and metabolite patterns is feasible as is measurement of other medicines and potential biomarkers of disease states. Research on use of novel biological matrices is receiving significant attention, especially new micro-sampling methods that involve finger-prick blood draw. Such techniques may provide patients with great autonomy around their care and could lead to more frequent or fuller characterization of drug exposure (Scuderi et al., 2020). In addition to blood samples for TDM other feasible and convenient specimens, including hair, saliva and ocular fluid are also being investigated (Zhang and Zhang, 2018).

While there is consensus around the need for monitoring of the calcineurin- and mTOR inhibitors, assessment of plasma levels of antimetabolites is often not performed. Wang et al. and Nourbakhsk et al. present their experience with measurements of mycophenolic acid (MPA) in kidney and heart transplant recipients, documenting large between-subject pharmacokinetic variability and the influence of frequently used concomitant medication on MPA exposure. Wang et al. confirmed a negative effect of concomitant proton pump inhibitor treatment on the bioavailability of mycophenolate mofetil dispersible tablets. These data support previous recommendations for more personalized treatment with MPA^3^. However, the optimal frequency of MPA TDM or which clinical events should prompt determination of MPA AUC need to be further established.

Another area of interest is measurement of intracellular concentrations of immunosuppressants in immune cells circulating in the blood (e.g., peripheral blood mononuclear cells (PBMC)). It is conceivable that intracellular concentrations hold actionable information, which might ultimately lead to further improvements in TDM. Sallustio has written an excellent review on the topic. The conclusion is that the verdict is still out. Available studies of tacrolimus largely describe a poor correlation between concentrations in the blood and in PBMCs but so far only identified the haematocrit value as a consistent determinant of distribution. Future studies should employ methods, which allow for elucidation of a more complex relationship including temporal delays in achieving equilibrium, and the role of specific drug transporters and drug metabolizing enzymes in addition to haematocrit.

TDM can also assist with checking patient compliance with therapy. Shi et al. identified patient adherence to immunosuppressant therapy as one of the most crucial factors for long-term allograft survival in a systematic review and meta-analysis. They suggested that clinicians should ideally establish a long-term intervention protocol to foster immunosuppressant adherence. To realize this goal, a multidisciplinary team-led, intervention approach combined with smartphone monitoring was recommended.

In summary, while TDM is routinely used for several immunosuppressant agents there are still much to learn in this field. Further research is required into optimal references ranges to target in different patient cohorts and under varying immunosuppressant combinations; how to best perform dosage adjustments based on exposure measurements and patient response; sources of pharmacokinetic variability associated with these agents; whether other novel mediums and methods could be used to better access drug exposure or immune response; and how to influence patient behaviour to ensure adherence with immunosuppressant treatments. With few new immunosuppressive medicines under development in the transplant field the continued pursuit of novel methods to better personalize the medicines we currently have is warranted.
